# Academic Goals and Parental Control in Primary School Children

**DOI:** 10.3390/ijerph17010206

**Published:** 2019-12-27

**Authors:** Benito León-del-Barco, Santiago Mendo-Lázaro, Silvia Iglesias Gallego, María-Isabel Polo-del-Río, Damián Iglesias Gallego

**Affiliations:** 1 Department of Psychology, Faculty of Teacher Training College, University of Extremadura, 10071 Caceres, Spain; silviaiglesiasgallego@gmail.com (S.I.G.); mabelpdrio@unex.es (M.-I.P.-d.-R.); 2 Department of Didactic of Musical, Plastic and Corporal Expression, Faculty of Teacher Training, University of Extremadura, 10071 Cáceres, Spain; diglesia@unex.es

**Keywords:** academic goals, parenting styles, parental control, primary education

## Abstract

Parenting styles have been used to explain the effects of family socialization on children’s learning skills. In this research, we have considered build an instrument for evaluating academic goals in the primary school stage, that allows us determine the relationships between the different types of goals and the different ways of establishing and policing the rules that the participants perceive from their parents. Those participating in this research were 550 pupils from of primary education. The Questionnaire on Academic Goals (QAG) has highly acceptable psychometric characteriztics. The analysis has shown the existence of four solid, well-defined factors. The relationships between the different types of goals and the different ways of establishing and policing the rules are verified. The pupils classified in the groups concerning the goals of social evaluation and reward were characterized by a more indulgent parenting style, determined by an absence of rules and limits for their children’s behavior. On the other hand, those pupils classified in the groups concerning the goals of learning and achievement were characterized by parents with an inductive style, determined by the use of reasoning and explanations towards their children in so far as the consequences of breaking the rules.

## 1. Introduction

The study of the different types of academic goals has traditionally considered those of learning and performance. The pupils in class become involved in a task and make an effort for two different reasons. On the one hand, they wish to acquire knowledge, increase their competence, dominate and enjoy the task; in short, they wish to learn and improve their skills, so we are talking of learning goals, also called proficiency goals or task-centered goals [1,2,3]. On the other hand, there are pupils who make an effort because they want to get good marks, advance in their studies and appear clever to others, thus avoiding being seen as incompetent; in short, they wish to demonstrate their value and their capacity and look for positive evaluations of their accomplishment, so we are talking of performance goals, also described as execution goals or ego-centered goals [1,2,3].

From this two-dimensional approach to a three-dimensional approach [4], and a 2 × 2 model [5,6], we have come to a 3 × 2 model [7]. Lower and Turner [8] pointed out that everyone feels the need to be successful and avoid failure. The resulting motivation usually pushes us to take risks in order to achieve the desired success or avoid certain situations

The three-dimensional model [4] considers the difference between the goals of approximation and avoidance (direction of the goal) as fitting within the goals of performance, while the 2 × 2 model [5,6] adds the difference between approximation and avoidance goals to the learning goals (goal orientation).

Finally, the 3 × 2 model [7,9] aims to improve the accuracy of the 2 × 2 model by considering various references, such as task, self, and other, in the evaluation of students’ competences (‘My goals in the exams of the subjects I am taking are…’). It would thus be possible to obtain task-based goals with the tendency either of approximation or avoidance (‘To have many correct answers’; ‘To avoid incorrect answers’), ego-based goals (‘Do better than I did in previous exams’; ‘Avoid doing worse than in previous exams’) and, finally, other-based goals, similarly with the approximation or avoidance orientation (‘Do better than my companions’; ‘Avoid doing worse than my companions’).

### 1.1. Research and Academic Goals

It is not easy to classify works of research into academic goals. A large majority relate goals to learning factors, including performance. Others look at how certain personal factors can determine a particular type of goal.

As for those studies that relate academic goals to learning factors, several research works demonstrate the positive correlations between learning goals and learning strategies [10,11]. Yet other research works relate academic goals to performance, to the strategic processing of information and the regulation of the learning process [1,12,13].

With respect to the personal factors, some authors sustain that students’ goals depend on personal variables, considering them to be stable constructs of a dispositional nature [14]. Such variables as the students’ perception of intelligence have been studied. Those students who believe intelligence depends on effort are more inclined towards learning goals than those who consider intelligence to be a stable characteristic, who generally prefer performance goals.

Relations between the goals of the 2 × 2 model [6] and the factors of the five degrees of personality model, a central theory in the study of personality traits [15], have also been analyzed. Other authors [16] find a strong relationship between the factors of conscience and kindness with the goals of learning approximation in a university sample. Similarly, the neuroticism factor was positively related with avoidance goals. In the same line of work with the five degrees of personality model and the 2 × 2 model, Chen and Zhang [17] found that personality is a better predictor than temperament.

Other authors have studied the relation between aggressive behavior and goals [18,19]. The relations between the different sociometric types (popular-preferred, rejected-aggressive, rejected-timid, and ignored-forgotten) and prosocial behavior with academic goals have also been examined in samples of adolescents in secondary education [20,21].

Other personal factors that have been studied include gender and age. Here the research has shown significant differences in favor of females as far as learning goals are concerned [22,23]. On the other hand, learning goals increase with age, while social evaluation goals decrease [24]. Recently, [25] have found that approximation goals positively predict satisfaction with life.

### 1.2. Present Study

This work has two aims: on the one hand, to build an instrument for evaluating academic goals in the primary school stage and, the principal one, to determine the relationships between the different types of goals and the different ways of establishing and policing the rules that the participants perceive from their parents.

As for the first objective, although the 2 × 2 and the 3 × 2 models both evaluate learning and performance goals, it is our opinion that there are other goals which are not directly related with learning and academic achievement, but which are still relevant in the context of our research. Between 10 and 12 years of age children undergo important cognitive changes; they develop abstract thought and the possibility of working with logical formal operations [26]. These new intellectual skills—as well as the influence of parents, peers, and teachers—are important in such aspects as socialization, building a self-image and in how children approach school work. Parents are the principal means to socialization and development and are the strongest force in the life of their children [27]. Similarly, the influence of peers is especially relevant for self-esteem and self-efficacy through reinforcement, role models and social comparison [28]. The teacher not only teaches subjects, she/he also evaluates and motivates the pupils, whose success is conditioned by the teacher’s perception of the pupils’ skills.

Pupils face learning tasks for academic reasons, but also for reasons of a prosocial nature; such research as that of Schneider, Ackerman, and Kanfer [29] support the importance of social goals, especially the desire for the approval of others. Hayamizu et al. [30] identify the goals of social reinforcement, related with the so-called social evaluation [31]. These goals are characterized by learning with the aim of obtaining social approval (from parents, teachers, and peers) and avoiding rejection. In addition to the orientation towards learning, there are other goals, such as the desire for a positive social evaluation and the approval of those others in a child’s life who are important, thus promoting involvement in school activities [13].

Children also make an effort and become involved in school activities for the prizes and rewards that are given, mainly by the parents: a new videogame, a smart phone, etc., as well as to avoid the curtailment of privileges: eliminating the smart phone’s internet connection, reducing the time allowed for watching television, etc. When parents reward or punish, their purpose is for the children to use the knowledge of the said prize or punishment to plan their actions. Along these lines, Alonso and Montero [31] proposed goals related with the attainment of rewards. These would not be directly related to learning, but can help and can involve both the attainment of prizes and the avoidance of any kind of punishment or loss of objects considered by the child as valuable [32].

As for our main objective, there is a minority group within academic goal research that analyses the influence of contextual factors, especially the classroom, on goals. They have studied the evaluation system, the classroom structure, the attitude of the teachers, etc. [1]. There has been little study of the influence of the family context on academic goals. Parenting styles have been used to explain the effects of family socialization on children’s learning skills, their identity and autonomy, key aspects for their development and learning. In particular, the contribution of Baumrind [33] has been influential in the study of parenting styles, outlining the different parenting styles which today are widely known in the scientific literature. Baumrind [34] later defined parenting styles with respect to the combination of two general factors that he called ‘demand’ and ‘sensibility-receptivity’.

Certain works of research have studied the existing relation between parenting styles and their influence on the educational sphere. For Spera [35], parenting styles would be related to the differences in the parents’ involvement in their children’s school life, their concern about the work their children do and their academic progress. Other works of research relate parenting practices to the pupils’ strategies aimed at academic success, self-esteem and academic performance. Inductive parenting practices are related with adaptive learning strategies, high self-esteem, and better performance [35,36,37,38,39]. However, there are no works that look into the influence on goals of the family context, in particular parental rearing patterns.

One problem with research into parenting styles is that parents often do not have a defined rearing style. Parenting styles are not exclusive and parents can have a main style, but with specific parenting practices from other styles. For this reason, in our research, we have focused on one dimension (control of norms) and our aim is, through a discriminant analysis, to analyze the relationships between the different types of goals (learning, performance, social evaluation, and reward) and the ways to establish and require compliance with parental norms. An inductive method, characterized by the use of reasoning and explanations concerning the consequences of not complying with the norms and of carrying out prohibited actions; an indulgent method, characterized by the absence of any norms or limits to behavior; and a rigid method of imposing norms, characterized by a high level of demands and punishment for not complying.

Similarly, we analyze how the different ways to establish and demand compliance with norms (Inductive-Father, Inductive-Mother, Indulgent-Father, Indulgent-Mother, Rigid-Father, and Rigid-Mother) present a greater power of discrimination or better quantification of the differences between the types of goals (learning, performance, social evaluation, and reward).

## 2. Materials and Methods

### 2.1. Participants

The sample of participants was made up of 550 pupils. Their average age was 10.73 years (SD = 0.632; range 10–12); 47.5% (*n* = 261) female and 52.5% (*n* = 289) male. The pupils were in fifth year (45.10%) and sixth year (54.90%) of Primary Education. The number of participants was determined by the number of pupils in the academic year 2016–2017, considering a sample error of 3% and a confidence level of 95.5%. The selection of pupils was carried out by means of a multi-stage sampling by clusters and random selection of the groups in the centers with several classes in the fifth and sixth years of primary education. The sampling by clusters was carried out by a random selection of four state schools.

### 2.2. Instruments

#### 2.2.1. Scale of Norms and Demands Children’s Version (ENE-H) [40]

This instrument has 3 factors: F1 inductive form, F2 rigid form and F3 indulgent form, which evaluate the way in which the parents establish and demand compliance with their norms. The first and second factors are made up of 10 items (F1Father (α = 0.79), F1Mother (α = 0.76); (F2Father (α = 0.77), F2Mother (α = 0.76)), while the third has 8 items (F3Father (α = 0.73), F3Mother (α = 0.74)). The response scale is the same as in the EA-H, the range of scores in the first two factors goes from 10 to 50, while the third factor’s scores go from 8 to 40. The pupil has to respond to the contents of each item according to his/her perception of the father’s or the mother’s educational style (e.g., ‘Before punishing me, he/she listens to my reasons’).

Why does the child’s perception of the way in which the parents establish and demand compliance with their norms interest us? Several studies have found a low coincidence between the opinions of the parents and their children concerning parenting practices [41,42]. Parents usually have their own perception concerning their parenting practices and it is sometimes biased due to a certain social desirability. The perception of the adolescent children has less bias, is more objective and may be an important predictor of their responses, more so than that of the parents.

#### 2.2.2. Academic Goal Questionnaire (AGQ)

This questionnaire is an adaptation of the Goals Questionnaire [30,43] translated to Spanish. The adaptation was done with the factorial analysis of [44] in mind, eliminating the items that were least saturated for each of the three factors, leaving only 15 of the original 20 items. On the other hand, we have added another 5 items to these 15 (five for each factor), 5 items which make up another new factor taken from the theory set out by [45]. The questionnaire is, therefore, made up of 20 items which look at the reasons why pupils study. The response scale is a Likert type scale with 5 degrees of frequency (1 = never, 2 = occasionally, 3 = sometimes, 4 = often and 5 = always).

### 2.3. Procedure

The study previously received the approval of the Ethics Committee of the University of Extremadura. The data were obtained from four state schools. We followed the ethical guidelines of the American Psychological Association [46] and informed consent forms were requested from both pupils and parents. The school councils of all the centers agreed the participation. No compensation of any kind was offered, their participation being totally voluntary. The instruments were completed in front of a trained interviewer and in the context of the classroom.

## 3. Results

### 3.1. Psychometric Characteriztics of the Academic Goals Questionnaire (AGQ)

The original sample (*n* = 550) was divided into two sub-samples extracted at random. The first (*n*^1^ = 300) was used to carry out the exploratory factorial analysis (EFA) and the second (*n*^2^ = 250) for the confirmatory factorial analysis (CFA). The internal consistency of the questionnaire measured using Cronbach’s Alpha index is 0.807. A factorial analysis was carried out to calculate the validity of the instrument’s construct. The Kaiser–Meyer–Olkin measurement of the sample’s adequacy offers a value of 0.838. Bartlett’s sphericity test is significant (Chi-square = 2506.161 and *p* < 0.001). The factor extraction model used is that of principal components with Oblimín rotation. The objective is to find a series of components that explain the maximum total variance of the original variables (Table 1).

The first factor, which we shall call ‘Learning’, explains 23.5% of the variance and refers to the intrinsic motivation, of competence and control, that pupils have with respect to school activities; for instance: ‘I study because I want to know new things’. It has a Cronbach’s Alpha of 0.825. The second factor, ‘Social evaluation’, explains 22% of the variance and evaluates the pupil’s goal with respect to experiencing the approval of both adults and peers, as well as avoiding rejection, for instance: ‘I study because I want others to see how clever I am’. The internal consistency is acceptable (Cronbach’s Alpha 0.814). The third factor, ‘Achievement’, explains 8.5% of the variance and refers to the goals related with the ego, the motivation of achievement and the fear of failure, for instance: ‘I study because I want to achieve a good social position in the future’. This has a Cronbach’s Alpha of 0.824. The fourth and final factor, ‘Reward’, explains 7.5% of the variance and refers to everything that implies prizes or rewards and avoiding punishments or the loss of valued situations, objects or possibilities; for instance: ‘I study because I want them to buy me a present’. This has a Cronbach’s Alpha of 0.830. The accumulated variance of the four factors is 61.5% and the internal consistency of the questionnaire has a Cronbach’s Alpha of 0.807.

A good practice when studying the validity of a questionnaire is to confirm the factorial structure found in the exploratory analysis with a confirmatory factorial analysis [47]. Given that the Exploratory Factorial Analysis (EFA) was not conceived to prove hypotheses or theories, we subjected the data to a Confirmatory Factorial Analysis (CFA) to check the following prior hypotheses: (a) number of factors and whether they are related to each other or are independent, and (b) with which factor or factors is each one of the variables related (weights).

In order to perform the estimations using the method of Maximum Likelihood [48], the linearity suppositions must be complied with and all the observed variables included in the model should have a normal distribution. Before the analysis to determine whether the sample complies with the normality criteria or not, we proceed with the detection of atypical values by applying the so-called Mahalanobis distance, using the option tests for normality and outliers of the AMOS program. Nevertheless, after eliminating the atypical scores, the data from the sample did not comply with the normality criteria, so we used the alternative method for estimating the parameters, the weighted least squares method.

In order to determine whether the model adequately adjusted to the data, the following goodness of fit values and indices have been used: Chi-square probability (χ^2^), degrees of freedom (CMIN/gL), comparative fit index (CFI), Tucker-Lewis index (TLI), root mean square error of approximation (RMSEA), and standardized root mean square residual (SRMR). Finally, in order to compare the different models, one with the other, we have used, as parsimony adjustment measures, the AIC (Akaike information criterion) and the BBC (Browne–Cudek fit criterion). The lowest values of the AIC and the BBC indicate a better fit with respect to the other alternative models.

The CFA is carried out with a sample of *n* = 250 using the 20 items of the QAG. Table 2 shows the statistics for the goodness of fit considering five models. In our hypothetical base model M1, model of four related factors, the items are loaded in their respective related hypothetical factors (F1 learning, F2 social evaluation, F3 achievement, F4 reward); M2, model of four independent factors, where the items are loaded in their respective independent hypothetical factors (F1 learning, F2 social evaluation, F3 achievement, F4 reward); M3, dichotomous model with two factors, where the items loaded in the dimensions of learning and achievement do so in a single latent factor (F1 academic), while the items loaded in the dimensions of social evaluation and reward also do so in a single latent factor (F2 incentive); M4, trichotomous academic model with three factors, where the items that define learning and achievement are loaded in a single latent factor (F1 academic), while the items loaded in the dimensions of social evaluation and reward do so in their respective hypothetical factors (F2 social evaluation and F3 reward); M5, trichotomous stimulant model with three factors, where the items that define learning and achievement are loaded in their respective hypothetical factors (F1 learning, F2 achievement), while the items that define social evaluation and reward are loaded in a single latent factor (F3 stimulant).

**Figure 1:**
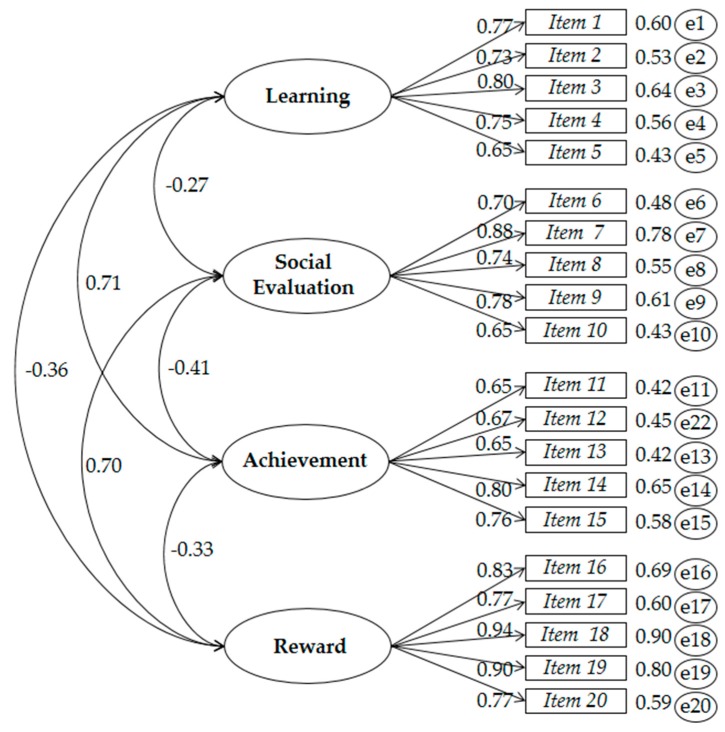
Model of four related factors of the Questionnaire of Academic Goals (QAG).

All the models have a significant value of χ^2^ (*p* < 0.05), but large sample sizes χ^2^ tend to be statistically significant so, from a practical perspective, it is more convenient to take into account the magnitude of the value of χ^2^ or χ^2^ divided by the degrees of freedom (CMIN/DF) than the statistical significance level; large values would correspond to a poor fit, while small values would correspond to a good fit. We discard the models: M2, M3, M4, M5. It is expected that the fitness indicators, CFI and TLI, should be greater than or equal to 0.90, a value that is not achieved in the said models. Neither are the values of the RMSEA and SRMR indicators achieved, which are expected to be lower than 0.08 and 0.06, respectively. The values that best fit what is expected can only be found in the model M1, shown in Figure 1; in addition, this model has lower values of AIC and BBC than the rest of the alternative models.

The internal consistency of the latent constructs was calculated by means of the compound reliability (CR) and the average variance extracted (AVE). For this research, it is desirable that the values of the CR should be equal to or higher than 0.7 and equal to or higher than 0.50 for the AVE. The factors, learning, CR = 0.876; AVE = 0.586, social evaluation, CR = 0.855; AVE = 0.544, achievement, CR = 0.877; AVE = 0.591 and reward, CR = 0.874; AVE = 0.582, corresponding to the model of four related factors, show evidence of reliability of the questionnaire.

### 3.2. Relations between Types of Goals and Ways of Establishing and Demanding Compliance with Parents’ Norms—Discriminant Analysis

We first of all calculated the value of the 75th percentile in the factors of the Questionnaire of Academic Goals (QAG): learning, social evaluation, achievement, and reward. The objective was to select those participants who had obtained the highest scores in the different factors. The descriptive and percentile scores for each of the factors were as follows: learning (M = 17.24, DT = 4.45, percentile 75:21), social evaluation (M = 10.91, DT = 5.34, percentile 75:15), achievement (M = 22.06, DT = 3.67, percentile 75:25), and reward (M = 10.40, DT = 5.23, percentile 75:14). Having selected the participants, they were classified into four subgroups, eliminating the subjects who had obtained scores higher than the percentile of 75% in two or more factors. That is, we only classified those subjects who obtained scores above the percentile of 75 in only one factor. Finally, four goal groups were established: learning (*n* = 48), social evaluation (*n* = 36), achievement (*n* = 62), and reward (*n* = 42).

Table 3 shows the means and standard deviations for each of the four goal groups into which the subjects were classified for the four factors of the ENE-H. All the goal groups obtained the highest average scores in the factors inductive mother and inductive father and the lowest average scores in the factors indulgent mother and indulgent father.

We then examined the possible existence of differences between the means of the four goal groups with respect to the scores in the factors of the ENE-H. To do so, we carried out an analysis of the variance (ANOVA). Significant differences were found between the four goal groups for the factors of the ENE-H: inductive, indulgent father, inductive mother, indulgent mother.

We later analyzed, using discriminant analysis, which factors of the ENE-H best explain these differences. In this study, as independent variables and predictors, we have used the six factors of the ENE-H (inductive father, rigid father, indulgent father, inductive mother, rigid mother, and indulgent mother), while we have used the different goal groups as dependent variables. In order to carry out the discriminant analysis, it is necessary for the linearity suppositions to be complied with, where all the observed variables included in the model follow a normal distribution (Kolmogorov–Smirnov test), as well as the equality of the variances-covariances (Box M test). We found *p* > 0.05 in all the contrasts, thus justifying the discriminant analysis.

Table 4 shows the structure matrix created in the discriminant analysis. The maximum number of discriminant functions or linear combinations is equal to one unit less than the number of groups assigned to the dependent variable. Which function has greater power of discrimination and will therefore be used to interpret the data? The analysis of the discriminant functions indicates that Function 1 is the one that presents the greatest power of discrimination in the four goal groups.

Function 1 explains a percentage of variance much higher than the other functions; it shows a greater canonical correlation and distance between the discriminated groups (Wilks’ Lambda closer to 0). In addition, the Chi-square analysis has the highest level of significance. Function 1 (% of variance = 70.4, canonical correlation = 0.635, Wilks λ: 0.458, χ^2^ = 46.859, *p* < 0.001). Thus, according to Function 1, the factor which has the greatest predictive capacity is the form indulgent mother (0.834). This is followed by indulgent father (0.763), inductive mother (−0.414) and inductive father (−0.346). In order to be able to interpret the relation of each factor with the different groups, it is necessary to know the means, and especially the sign, of the functions in the centroids of the goal groups. The group achievement = −0.760, social evaluation = 0.664, learning = −0.459, while reward = 1.144.

Finally, Table 5 shows that the discriminant canonical function obtained allows for the correct classification of 66.7% of the social evaluation group, 60% of the reward group, 54.2% of the achievement group and 40% of the learning group. The average gains in prediction were above the 25% which could be expected by chance in all the four categories of goals (achievement, social evaluation, learning, and reward).

## 4. Discussion

As for our first objective, it can be said that the Questionnaire of Academic Goals (QAG) has some very acceptable psychometric characteristics, good internal consistency, and sufficient evidence of validity and reliability. The factorial fitness measurements of Kaiser–Meyer–Olkin and Bartlett’s test confirm that the performance of the factorial analysis makes sense. The analysis carried out has demonstrated the existence of four solid, well-defined factors upon which we base the construction of the scales and which explain 61.5% of the total variance. The weights or loads of the items that define the four factors have values over 0.50. For Costello and Osborne [49], when a factor is defined by 4–5 items with weights of over 0.50, then this is a solid factor with practical relevance. Finally, the questionnaire was subjected to a confirmatory factorial analysis where five different factorial structures were put to the test. The values that best fitted what was expected were to be found in the model of four related factors. This model showed a better fit to the data than the rest of the models subjected to the confirmatory analysis, including the model of four independent factors. The models with the worst fit were those of four independent factors, trichotomous stimulant and trichotomous academic. The dichotomous model had the second best fit, after the model of four related factors.

The correlations between the factors ‘learning’ and ‘achievement’ are high and statistically significant (*p* < 0.01), as are the correlations between the factors ‘social evaluation’ and ‘reward’. The students who pursue learning goals also pursue achievement goals, they are not exclusive. These students approach learning tasks for academic reasons. Similarly, the students who pursue social evaluation goals also pursue reward goals and they are characterized by the desire for prizes and the approval of those who are important to them and who encourage involvement in school activities [13].

The confirmatory analysis has shown low inverse correlations between the factors ‘social evaluation’ and ‘reward’ with those of ‘learning’ and ‘achievement’. To summarize, the students who approach learning tasks looking for prizes and the approval of others do not usually have other reasons, such as increasing their skills, dominating and enjoying a task, improving their capabilities (learning goals) or getting good marks, advancing in their studies, wanting to appear clever to their peers… (achievement goals). The latter are not directly related with learning but they may help [32].

Many works of research have studied the existing relationship between parenting styles and their influence on the educational sphere, mainly students’ academic performance [35,36,38,39]. However, there are not many studies that relate parenting styles to children’s academic goals. The principal aim of this work is to analyze which ways of establishing and demanding compliance with the norms (inductive father, inductive mother, indulgent father, indulgent mother, rigid father, and rigid mother) influence the different goal types (learning, social evaluation, achievement, and reward).

The results obtained demonstrate the existence of differences between the four goal groups, ‘social evaluation’, ‘reward’, ‘learning’, and ‘achievement’, in the ways of demanding compliance with the norms. As for the results obtained in our research, a series of questions arise. As for the results obtained in our research, a series of questions arise.

Which factors best distinguish or quantify the differences between the goal types? The said factors are, in order, indulgent mother, indulgent father, inductive mother, and inductive father. In short, the pupils classified in the goal groups of social evaluation and reward are characterized by a parenting style of an indulgent form, determined by the absence of norms and limits on their children’s behavior. On the other hand, those pupils classified in the goal groups of learning and achievement are characterized by parents with an inductive style, determined by the use of reason and explanations for the children of the consequences of breaking the rules and of carrying out prohibited actions. In this sense, studies such as those of Chan and Chan [50] conclude that the students’ achievement orientation is related with this parenting educational style.

Why, in both situations, is it always the indulgent or inductive mother who has the greatest influence over goals? With respect to the differences between fathers and mothers, several studies state that there is an attenuation of the differences in parental care depending on the gender of the parent [51,52]. During the first years of a child’s life, it is the mother whose presence, speech, and demands are most felt, much more than the father’s [53]. Research has shown the greater involvement of mothers as opposed to fathers in the rearing of children. Laible and Carlo [54] found that mothers are more affectionate and favor the autonomy of their children more. The educational practices of the mothers are perceived as being more positive than those of the fathers, since they achieve higher scores in affection and lower scores in rejection, coinciding with Bersabé et al. [41] and Garcia et al. [55].

Why does the inductive form of establishing and demanding compliance with norms predict the learning and achievement goals in primary school children? The establishment of rules and clear limits, and the demand that they be complied with in the family context, is one of the primordial functions of the parents as far as their children are concerned [56]. The inductive style, characterized by clear and reasoned rules on the parents’ part increases the children’s capacity to act autonomously and responsibly [53,57]. Parents who demand a certain level of control and inductive discipline from their children, in doing so, encourage greater levels of emotional security, maturity, internalization of norms, acquisition of social and cognitive competences, as well as a high motivation towards achievement [58,59,60]. In the academic sphere, their goals are oriented towards the acquisition of knowledge, an increase in competences and progress in their studies, that is to say, towards learning and improving their own skills, which is a characteristic of the learning and achievement goals.

Why does the indulgent form of establishing and demanding compliance with norms predict the goals of social evaluation and reward? The parents who practice this form do not establish rules or limits to their children’s behavior, liberating themselves from the need for parental control, that is to say, from restrictions and punishments. This indulgent form would be related with an extrinsically motivational orientation [39,58] and generates dependent children who are not very committed to participating in school, with hardly any motivation or capacity for effort, as well as a lack of discipline [61]. All this leads us to believe that they will be students with goals that depend on social evaluation and reward by their parents.

We have to say that this study does have several limitations, such as the use of self-reporting by the participants in both the evaluation of academic goals and the ways the parents have of establishing and demanding compliance with norms. We believe it would be important to have other informants besides those who are directly involved. We consider that teachers are in a privileged situation for analyzing academic goals as they can evaluate them on a daily basis in class. Similarly, within a bidirectional model of parent–children relations, it would be convenient to evaluate the ways in which parents establish and demand compliance with norms from the point of view of the parents. Although the majority of research works concerning parenting styles fit the two dimension model (affection-control) of Maccoby and Martin [62], there are other dimensions, such as the promotion of autonomy, revelation, humor, or psychological control, which should be kept in mind for future research, but the dimension of affection is the most important. The differences in the number of cases in each of the goal groups should lead us to consider the results with caution, that is until we are able to increase this number.

## 5. Conclusions

Finally, we believe that the parents form the principal means of socialization and development of everyone, from a very early age. The role of the family in a person’s psychosocial development is undisputed; the parents are the most powerful force in their children’s lives. For Rodrigo and Palacios [63], the parents’ view of education, interpersonal relationships and parenting styles are determinant factors in their children’s development. It would be useful to design intervention and formation programs with families in order to increase the parents’ feeling of satisfaction and competence concerning their duties and responsibilities as parents, as well as to achieve an improvement in parent–child relations. The formation of the parents is a part of the children’s education, as well as being a method to promote their development and learning in school.

## Figures and Tables

**Table 1:** Factorial analysis of the instrument: Principal components with Oblimín rotation.

M	DT	Items of the Instrument	Factor			
			1	2	3	4
3.37	1.06	1. I study because solving problems is interesting.	0.788			
3.53	1.20	2. I study because I enjoy discovering how much I have improved.	0.764			
3.92	1.07	3. I study because I want to know new things.	0.781			
2.97	1.25	4. I study because I like the challenge of difficult problems.	0.731			
3.47	1.24	5. I study because I feel curiosity.	0.751			
1.76	1.17	6. I study because I want my classmates to notice me.		0.804		
2.20	1.48	7. I study because I don’t want my classmates to make fun of me.		0.746		
2.37	1.58	8. I study because I don’t want the teacher to hate me.		0.670		
2.20	137	9. I study because I want the others to see how clever I am.		0.802		
2.38	1.43	10. I study because I like getting better marks than my classmates.		0.744		
4.73	.74	11. I study because I want to have good marks.			0.795	
4.54	.87	12. I study because I want to be proud of getting good marks.			0.772	
3.94	1.21	13. I study because I want to go on to higher education.			0.632	
4.57	.88	14. I study because I want to have a good job in the future.			0.858	
4.29	1.01	15. I study because I want to have a good social position in the future.			0.809	
1.87	1.22	16. I study because I get presents if I get good marks.				0.803
1.57	1.10	17. I study because they buy me presents when I do the work well.				0.779
2.55	1.61	18. I study because I don’t want to be punished without playing or going out.				0.770
2.68	1.62	19. I study because I don’t want them to take away my favourite activity.				0.717
1.73	1.20	20. I study because I want to get a present.				0.788
Percentage of variance explained (Total 61.5%)			23.5%	22%	8.5%	7.5%
Cronbach’s Alpha			0.825	0.814	0.824	0.830

**Table 2:** Goodness of fit indices of the proposed models.

Models	χ^2^	CMIN/χ^2^	CFI	TLI	RMSEA	SRMR	∆CMIN/χ^2^	AIC	BCC
M1: Four related factors	392.87	2.455	0.922	0.907	0.070	0.058		492.86	500.82
M3: Dichotomous	468.81	2.859	0.897	0.881	0.081	0.089	0.404	560.81	568.12
M4: Trichotomous Academic	518.71	3.106	0.882	0.865	0.086	0.077	0.651	604.71	611.55
M5: Trichotomous Stimulant	558.26	3.343	0.868	0.850	0.091	0.078	0.888	644.26	651.10
M2: Four independent factors	1085.97	6.388	0.692	0.652	0.137	0.160	3.933	1165.97	1172.35

**Table 3:** Means and standard deviations for ENE-H factors with respect to the different goal groups into which the participants were classified.

Scale of Norms and Demands: Children’s Version (ENE-H)		Goal Groups				ANOVA	
		Learning	Social Evaluation	Achievement	Reward	*F*	*p*
Father	Inductive M (DT)	43.20 (4.26)	36.92 (4.90)	41.37 (6.77)	39.06 (6.98)	2.860	0.044
	Rigid M (DT)	23.33 (6.68)	27.25 (6.86)	25.42 (5.68)	29.27 (8.87)	2.022	0.120
	Indulgent M (DT)	14.13 (5.11)	15.17 (2.52)	12.75 (4.70)	20.87 (11.44)	4.772	0.005
Mother	Inductive M (DT)	43.73 (4.30)	37.33 (5.10)	42.62 (4.98)	40.27 (6.32)	4.135	0.010
	Rigid M (DT)	23 (7.11)	28.33 (6.03)	26.83 (6.53)	29.87 (9.50)	2.372	0.079
	Indulgent M (DT)	13.73 (4.28)	15.25 (2.93)	11.62 (3.17)	18.40 (4.48)	10.509	0.000

**Table 4:** Structure matrix. Variables ordered by the size of correlation with the discriminant function.

Variables	Functions		
Factors ENE-H	Function 1	Function 2	Function 3
Form Indulgent Mother	0.834 *	0.431	−0.109
Form Indulgent Father	0.531 *	0.411	0.280
Form Inductive Mother	−0.414 *	0.651	0.043
Form Inductive Father	−0.346 *	0.525	−0.170
Form Rigid Mother	0.293	−0.231	0.729
Form Rigid Father	0.326	−0.092	0.527

* Greatest absolute correlation between each variable and the discriminant function.

**Table 5:** Results of the classification using the discriminant function

Grouping Variables	Predicted Group			
Academic Goals	Achievement	Social Evaluation	Learning	Reward
Achievement	54.2%	8.3%	29.2%	8.3%
Social Evaluation	8.3%	66.7%	8.3%	16.7%
Learning	40%	13.3%	33.3%	13.3%
Reward	6.7%	13.3%	20.0%	60%

Classified correctly 53% of the cases originally grouped together.
